# Supplementary material for: Effect of Dietary Supplementation with Omega-3 Fatty Acid on the Generation of Regulatory T Lymphocytes and on Antioxidant Parameters and Markers of Oxidative Stress in the Liver Tissue of IL−10 Knockout Mice
Source: Nutrients. 2024 Feb 24;16(5):634. doi: 10.3390/nu16050634 (PMC10934328; doi:10.3390/nu16050634)
Supplement: Supplementary file 1 [file nutrients-16-00634-s001.zip › nutrients-2801979-supplementary.pdf]

Supplementary figure S1. **Analysis strategy for obtaining regulatory T cells.** Cells from the lymph node of animals *IL-10$^{-/-}$ +O3; IL-10$^{-/-}$; WT+O3 and WT.*

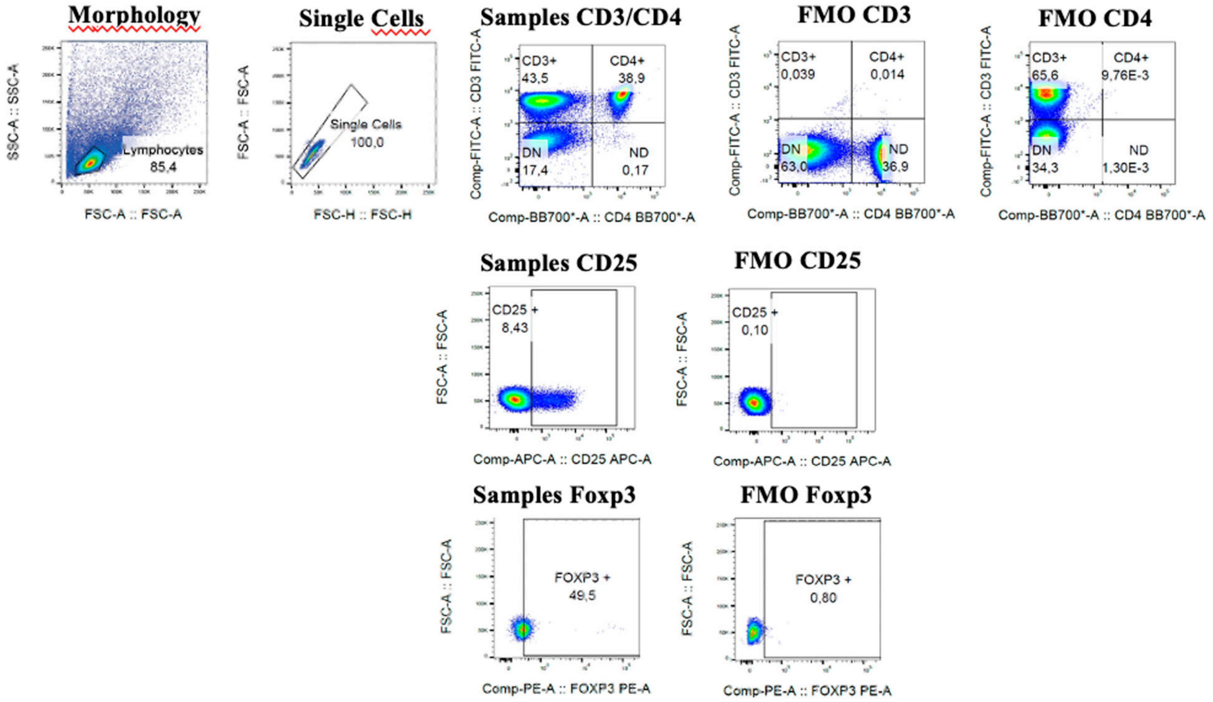

Supplementary figure S2. Assessment of IL-6 production in mesenteric lymph node culture supernatant after 48 hours of culture with addition of ConA. Groups *IL-10$^{-/-}$ +O3; IL-10$^{-/-}$; WT+O3 e WT.*

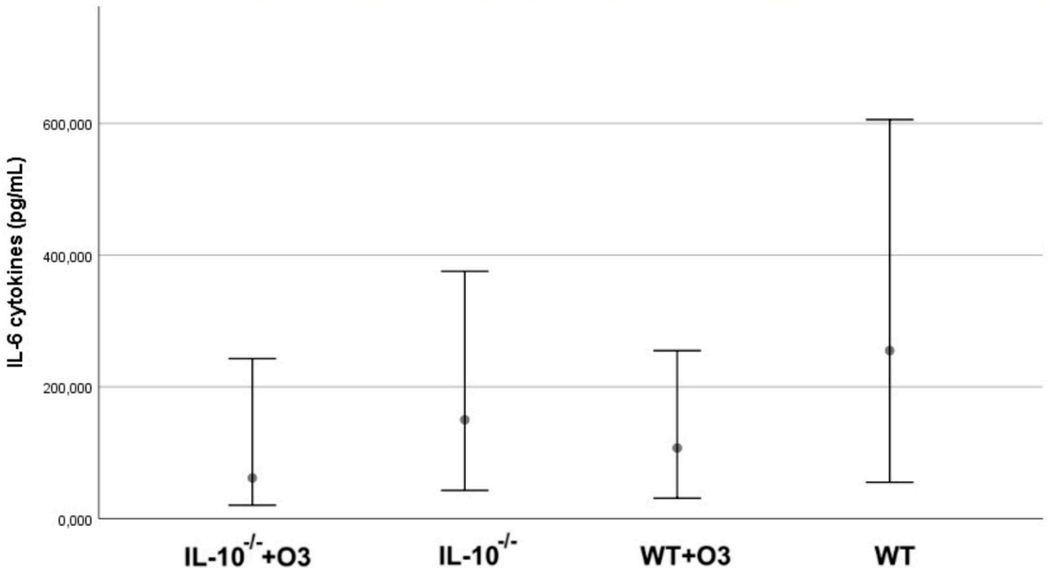

Supplementary Table S1. Median IL-6 production in mesenteric lymph node culture supernatant after 48 hours of culture with addition of ConA

Variables	IL-10$^{-/-}$+O3		IL-10$^{-/-}$		WT+O3		WT		Kruskal-Wallis p-value
	Median	IQR	Median	IQR	Median	IQR	Median	IQR	
IL-6	61.76	41.40-177.63	150.13	73.76-273.31	107.22	34.88-224.54	255.17	65.44-496.16	0.162

Note: IQR means interquartile range. *Means statistical significance (p<0.05). The Bonferroni post test is not applicable when the Kruskal-Wallis p-value is >0.05.

Supplementary Table S2. Muscle weight between groups *$IL\text{-}10^{-/-}$ +O3; $IL\text{-}10^{-/-}$; WT+O3 e WT*

Variables	$IL\text{-}10^{-/-}$+O3		IL-10		WT+O3		WT	
	Median	IQR	Median	IQR	Median	IQR	Median	IQR
Weight gastro	0.14	0.13 - 0.14	0.13	0.10 - 0.15	0.12	0.11 - 0.14	0.11	0.10 - 0.12
Weight tibialis	0.04	-	0.04	0.03 - 0.04	0.04	-	0.04	-
Weight edl	0.01	-	0.01	-	0.01	-	0.01	-
Weight soleus	0.01	-	0.01	-	0.01	-	0.01	-

Note: IQR means interquartile range. The empty IQR cells means absence of data variation.

Supplementary Table S3. Muscle GPx function between groups *$IL\text{-}10^{-/-}$ +O3; $IL\text{-}10^{-/-}$; WT+O3 e WT*

Variables	$IL\text{-}10^{-/-}$+O3		IL-10		WT+O3		WT		Kruskal-Wallis p-value
	Median	IQR	Median	IQR	Median	IQR	Median	IQR	
GPx muscle	158.1	140.1 - 161.7	148.4	132.5 - 157.3	150.3	135.3 – 190.4	149.9	139.1 - 180.7	0.726

Note: IQR means interquartile range.
